# Expression of Chondrogenic Potential Markers in Cultured Chondrocytes from the Human Knee Joint

**DOI:** 10.1177/19476035241241930

**Published:** 2024-04-14

**Authors:** John-Peter Bonello, M. Yat Tse, Trevor J. G. Robinson, Davide D. Bardana, Stephen D. Waldman, Stephen C. Pang

**Affiliations:** 1 Department of Biomedical and Molecular Sciences, Queen’s University, Kingston, ON, Canada; 2 Division of Surgery, Kingston General Hospital, Kingston, ON, Canada; 3 Department of Chemical Engineering, Toronto Metropolitan University, Toronto, ON, Canada

**Keywords:** osteoarthritis, human knee joint, cultured chondrocytes, chondrogenic potential markers, age, sex

## Abstract

**Objectives:**

While substantial progress has been made in engineering cartilaginous constructs for animal models, further research is needed to translate these methodologies for human applications. Evidence suggests that cultured autologous chondrocytes undergo changes in phenotype and gene expression, thereby affecting their proliferation and differentiation capacity. This study was designed to evaluate the expression of chondrogenic markers in cultured human articular chondrocytes from passages 3 (P3) and 7 (P7), beyond the current clinical recommendation of P3.

**Methods:**

Cultured autologous chondrocytes were passaged from P3 up to P7, and quantitative polymerase chain reaction (qPCR) was used to assess mRNA expression of chondrogenic markers, including collagen type I (COLI), collagen type II (COLII), aggrecan (AGG), bone morphogenetic protein 4 (BMP4), transcription factor SOX-9 (SOX9), proteoglycan 4 (PGR4), and transformation-related protein 53 (p53), between P3 and P7.

**Results:**

Except for AGG, no significant differences were found in the expression of markers between passages, suggesting the maintenance of chondrogenic potential in cultured chondrocytes. Differential expression identified between SOX9 and PGR4, as well as between COLI and SOX9, indicates that differences in chondrogenic markers are present between age groups and sexes, respectively.

**Conclusions:**

Overall, expression profiles of younger and male chondrocytes exhibit conversion of mature cartilage characteristics compared to their counterparts, with signs of dedifferentiation and loss of phenotype within-group passaging. These results may have implications in guiding the use of higher passaged chondrocytes for engineering constructs and provide a foundation for clinical recommendations surrounding the repair and treatment of articular cartilage pathology in both sexes.

## Introduction

With limited repair capacity, articular cartilage is susceptible to damage from trauma, and it can progress to long-term diseases such as osteoarthritis (OA) that may require knee replacement.^1^ Brittberg *et al.*^2^ initiated the use of cultured autologous chondrocytes from the knee joint of patients for the treatment of deep cartilage defects. Their results indicated that cultured chondrocytes behaved as mesenchymal stem cells (MSCs) and have the potential to repair cartilaginous injuries.^3^ In fact, autologous MSCs for cartilage repair are an expanding topic in tissue engineering literature, particularly successful in animal models including rabbits^4,5^ and sheep.^6,7^ Recently, the chondrogenic potential markers (CPMs) of passaged autologous bone marrow–derived MSCs were tested. It demonstrated greater genomic instability and indicators of senescence at P3 when compared to bone marrow mononuclear cells.^8^ Markers of cell cycle, DNA replication, and mismatch repair properties, were downregulated in bone marrow MSCs at P3. As a result, researchers and clinicians recommend the implementation of autologous MSCs to be used before P3. However, these observations need to be verified in human cartilage samples, one of the objectives of this study.

**Figure 1.**
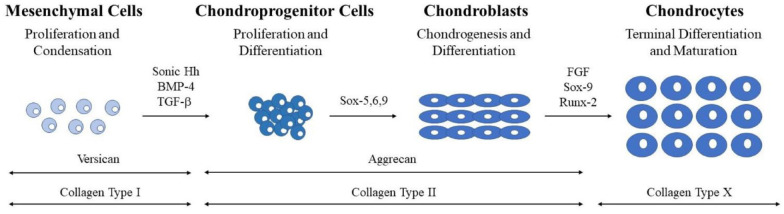
Sequence of events during chondrocyte development. Stages categorized by cell type and represented schematically. Transcription and differentiation factors are listed above the arrows between the histological representations of the cells. ECM proteins are listed above double-headed arrows at the base of the figure.

During the process of MSC recruitment, migration, proliferation, and condensation, there are a plethora of transcriptional factors that play an important role in regulating chondrogenesis (**Fig. 1**). Many of these factors have not been fully evaluated in cartilaginous regeneration. In the developing limb bud, a signaling molecule called sonic hedgehog first initiates chondrogenesis.^9^ Sonic hedgehog signaling then induces bone morphogenetic proteins (BMP) and directs MSCs toward chondrogenic differentiation.^10^ Once the lineage is determined, a nuclear transcription factor known as sex-determining region Y (SRY)-box 9 (Sox9) protein becomes expressed.

It is well known that type II collagen is a main component of cartilage.^11^ More specifically, Valcourt *et al.*^12^ demonstrated that transforming growth factor (TGF)-β1 is associated with the expression of procollagen form COLII alpha-1. Acting as one of the earliest markers of chondrogenesis, Sox9 is integral for the expression of the type II collagen (COLII) gene, and the suppression of runt family transcription factor 2 (Runx2).^13,14^ Two additional Sox family members, L-Sox5 and Sox6 are involved in the initial regulation of chondrogenesis. Working in concert, the 3 Sox transcription factors activate the expression of several cartilage-specific genes in addition to COLII, with the goal of achieving mature chondrocytic phenotype.^15^ As chondrogenesis is also an active process in the formation of bones, articular cartilage bound chondrocytes must also be under strict regulation by positive and negative feedback factors. Specifically, Sox9 negatively regulates maturation of early chondrocytes toward osteogenic differentiation, whereas L-Sox5 and Sox6 will prevent premature hypertrophy.^10^

Terminal differentiation of chondrocytes marks the final, crucial step for mature cartilage development. A leucine zipper protein, cMaf, is expressed in late chondrocytes.^16^ At this stage, hypertrophy of chondrocytes is common, marking terminal differentiation and causing cells to secrete angiogenic factors such as vascular endothelial growth factor (VEGF).^17^ Primarily, these secretions are restricted to chondrocytes that will become ossified, or the few that lay close to subchondral bone. Deviations in angiogenic factors or their receptors typically result in a deficit in the growth and replacement of both cartilage and bone, tagging angiogenic factors as a key component of normal development and ossification.^17^

In addition, the extracellular matrix (ECM) plays an integral role in determining and regulating chondrocyte development. The compositional changes of the stem cells’ extracellular environment, known as the stem cell niche, provide differing external signals for gene expression determining cell fate.^18,19^ Signaling pathways within the ECM such as BMP and fibroblast growth factor (FGF) are activated to dynamically control their composition. Specifically, the bioactivity of growth factors such as transforming growth factor β (TGF-β) and tumor necrosis factor α (TNF-α) are modulated by ECM components, resulting in proteolytic changes to the environment and phenotype.^20^ The presence of COLII and aggrecan (AGG) characteristically mark differentiated chondrocytes, with deviations in expression or content acting as a viable measure of degradation or alterations in differentiation.^21^

The objective of this research was to refine the isolation protocol of chondrocytes and further test the CPMs of these human articular cells for tissue engineering. Isolated from injured human knee joints, healthy chondrocytes from varying participants were cultured, passaged, and tested for markers of differentiation with the aim of enhancing clinical recommendations and determining differences in age and sex.

## Materials and Methods

### Participants and Surgical Procedures

The experimental protocol for this project involved participants from the Division of Orthopaedic Surgery at Kingston General Hospital, and it was approved by the Queen’s University Health Sciences Affiliated Teaching Hospitals Research Ethics Board (Protocol #: SURG-343-16).

Six participants were included in the analysis of this study, stratified by both sex and age. Specifically, 2 female and 1 male participants under the age of 40 were included in the one category, and conversely, 2 male and 1 female over the age of 40 in another. All participants underwent surgery to treat an articular cartilage surface injury of the knee joint.

### Chondrocyte Isolation, Culture Condition, Passaging, and Freeze–Thaw Procedure

After injured edges were removed, cartilage samples were collected from healthy articular cartilages. The trimmed cartilage tissue was diced into 2 to 4 mm^3^ cubes ( **Figure S-1** , Supplemental data). Tissue blocks were treated with 100 μg/mL streptomycin, 100 units/mL penicillin, 0.25 μg/mL amphotericin B, 25 mM HEPES, and 0.5% (w/v) protease in 20 mL of chondrocyte growth medium (Lonza Walkersville Inc., Walkersville, MD, USA) for 90 minutes. Growth medium was used to wash the samples 3 times before an 18-hour digestion in 15 mL of 0.15% collagenase A (w/v; Roche Diagnostics Canada, Laval, QC, Canada) in growth medium. Digestion protocols were carried out at 37°C with 95% humidity and 5% CO_2_. Once the digestion sequence was completed, tissues were passed through a 200-mesh filter (Sigma-Aldrich) to remove undigested material, and the filtrate centrifuged at 600*g* to collect pelleted chondrocytes. Trypan blue exclusion test was used to evaluate cell viability (~90%-95%).

Isolated chondrocytes were cultured and passaged according to the published protocol in calves^22^ and humans^23^ with minor modifications. Briefly, isolated chondrocytes were cultured in T25 flask at density of 5 × 10^5^ cell/mL of high glucose Dulbecco’s Modified Eagle’s Medium (DMEM), containing 10% fetal bovine serum (FBS; HyClone Lab Inc., Logan, Utah, USA) and 1× antibiotic-antimycotic solution. Sample lots of FBS were evaluated for the support of human chondrocyte growth prior to purchase. Cell adhesion was assessed 24 hours later. Confluent monolayer chondrocytes were passaged, frozen, and thawed using previously published methods.^24,25^

### Cell Collection, RNA Isolation, and cDNA Transcription

**Figure 2.**
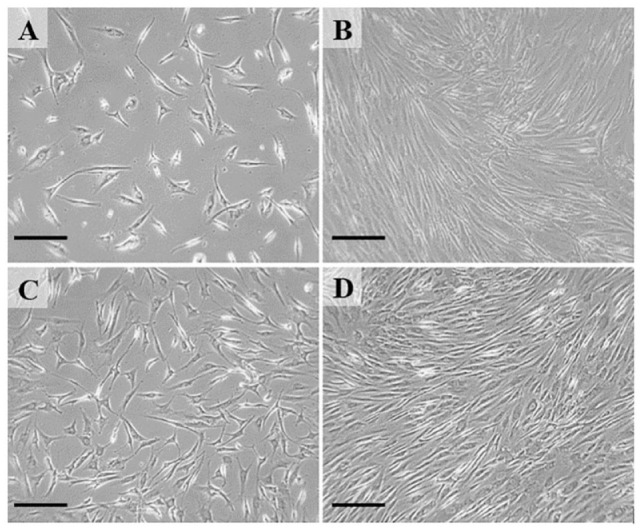
Phase contrast microscopy illustrating chondrocytes in culture. Panel **A** presents a P3 cell culture at ~40% confluence, with pseudopodia indicating cells are spreading out and adhering to the plate. Panel **C** presents a P7 cell culture at ~50% confluence, mimicking the pseudopodia adhesion seen in P3. Panel **B** and **D** present a P3 and P7 cell culture at confluence, respectively. Scale bar = 50 µm.

Cultured chondrocytes were maintained to either P3 or P7, and harvested at confluence for further evaluation (**Fig. 2**). At harvest, cells were washed with Hank’s Balanced Salt Solution, scrapped from culture flask, dislodged cells collected, and centrifuged to collect the cell pellet for RNA isolation.

Total RNA was isolated using a combination of Trizol (Tri Reagent, Molecular Research Center, Inc., Burlington, ON, Canada) and a high pure RNA isolation kit (Roche Scientific Co., Laval, QC, Canada). All wash and elution steps were carried out according to manufacturer’s instructions. RNA was measured using Nano-Drop 2000 spectrometer (Thermo Scientific, Wilmington, DE, USA).

Total RNA (100 ng) was reverse transcribed into cDNA using qPCRBIO cDNA synthesis kits (PCR Biosystems Inc., Wayne, PA, USA) with minor modifications. Briefly, sample RNA was pipetted to 15 µL of water before the addition of 4 µL of a 5× cDNA mix, containing oligo dT primers, and random hexamer deoxynucleotide triphosphate (dNTP) then used for the reaction. One µL of 20× RTase was added, containing a mixture of avian myeloblastosis virus (AMV) and RNase inhibitors. The reaction mixture solution was incubated at 42°C for 30 minutes.

### Primer Design and qPCR Protocol

Oligonucleotide primers for collagen type I (COL1), collagen type II (COLII), and AGG genes were previously designed by Robinson^23^ (**Table 1**). In addition, new oligonucleotide primers were designed for BMP4 and SOX9 using the Primer Design 2.01 software (Scientific & Educational Software, Cary, NC, USA) with published mRNA sequences from the NIH GenBank (National Center for Biotechnical Information, Bethesda, MD, USA; www.ncbi.nlm.nih.gov/Genbank). The remaining primers for p53, PGR4, and glyceraldehyde-3-phosphate dehydrogenase (GAPDH) were purchased as prevalidated primer sets from Integrated DNA Technologies, Inc. (Coralville, Iowa, USA).

**Table 1.** Summary of Primer Set Sequences.

Primer	Primer Pair Sequence	Primer Melting Temperature (°C)	Transcript Product Size (bp)	Efficiency	Product Melting Temperature (°C)
COLI	*Forward*: 5’-CGTCCTGGTGAAGTTGGTC-3’	*Forward*: 68	162	1.765	85
	*Reverse*: 5’-AGCCTCTCTCTCCTCTCTGA-3’	*Reverse*: 66			
COLII	*Forward*: 5’-ACGTCCAGATGACCTTCCTA	*Forward*: 67	178	1.911	83
	*Reverse*: 5’-GTACGTGAACCTGCTATTGC-3’	*Reverse*: 66			
AGG	*Forward*: 5’-CTACGACGCCATCTGCTAC-3’	*Forward*: 67	288	1.962	84
	*Reverse*: 5’-GGCCTCTCCAGTCTCATTC-3’	*Reverse*: 67			
BMP4	*Forward*: 5’-CACTGGTCTTGAGTATCCTG-3’	*Forward*: 63	296	1.840	88
	*Reverse*: 5’-AGTAGTCGTGTGATGAGGTG-3’	*Reverse*: 63			
SOX9	*Forward*: 5’-GGAGACTTCTGAACGAGAGC-3’	*Forward*: 67	123	1.534	89
	*Reverse*: 5’-TTCTTCACCGACTTCCTCC-3’	*Reverse*: 67			

Levels of mRNA expression were measured using the standard-curve method for each gene with GAPDH as a reference gene.^23^ All qPCR was performed using the LightCycler^®^ 480 system II (Roche Scientific, Laval, QU, Canada), and all samples were run in triplicates.

### Primer Verification

To ensure a single product was amplified during PCR, melt curve analyses were conducted for each primer set. The first derivative of the fluorescence versus temperature melt curves displays a Gaussian distribution; exhibiting a single peak that falls above 80°C. Typically, the primer dimers melt at temperatures between 70°C and 75°C, making them easily identifiable in the curve. All primer sets produced a Gaussian melt curve; however, COLII fluorescent expression levels were below the threshold needed for curve analysis.

### Statistical Analyses

Data were standardized by taking a ratio of the target gene expression over the expression of GAPDH. All statistical analyses were performed, and graphs plotted using Prism 6.0 Software (GraphPad Software Inc., La Jolla, CA, USA). Target gene mRNA expressional data were compared by 2-way analysis of variance (ANOVA) with multiple comparisons using Tukey’s *post hoc* test. Data are presented as mean ± standard error of the means (SEM). Values of *P* ≤ .05 were considered statistically significant.

## Results

### Passage Comparison of CPM Gene Expression

**Figure 3.**
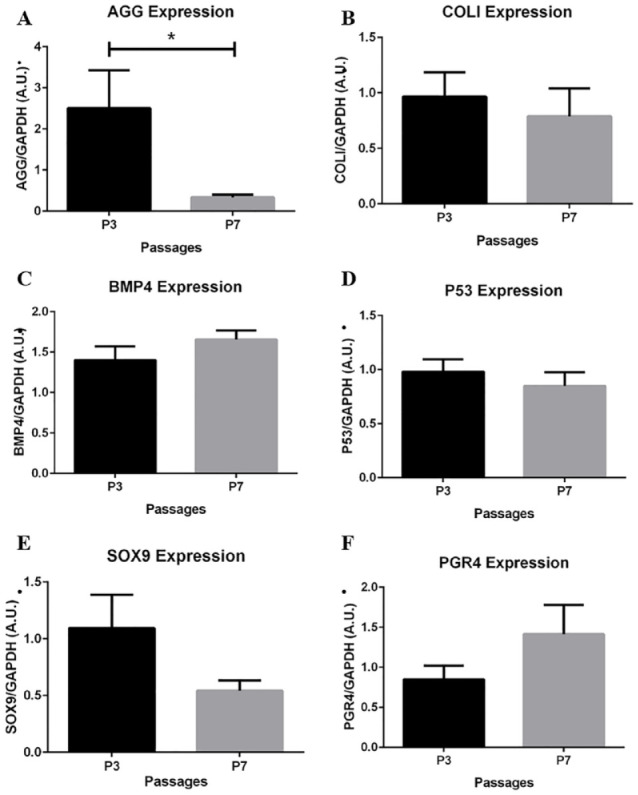
Passage comparison of chondrogenic potential marker (CPM) gene expression. (**A**) AGG expression is significantly decreased in P7 compared to P3 chondrocytes (7.4-fold change; *P* < .05). The remaining genes maintained a relative amount of expression from P3 to P7, with no significant differences (**B-F**). However, notable differences can be observed in SOX9 (**E**) and PGR4 (**F**) expression. Data are presented as the mean ± SEM; n = 6.

Each of the primer sets designed and implemented in the study were grouped by passage and tested for differences in relative gene expression. **Figure 3A** shows a 7.4-fold decrease in AGG expression from P3 to P7.

The remaining genes maintained a relative amount of expression from P3 to P7, with no significant differences. Specifically, comparing P3 to P7, a 2-fold decrease in SOX9 expression (**Fig. 3E**; *P* = .1) and a 1.6-fold increase in PGR4 expression (**Fig. 3F**; *P* = .2) were calculated.

### Age Comparison for CPM Gene Expression

Each chondrocyte culture and resulting gene expression profile underwent subgroup analysis by age comparing under (<40) or over (>40) 40 years. Significant changes in AGG, SOX9 and PGR4 expression demonstrated dedifferentiation of chondrocytes from patients <40 compared to >40.

**Figure 4.**
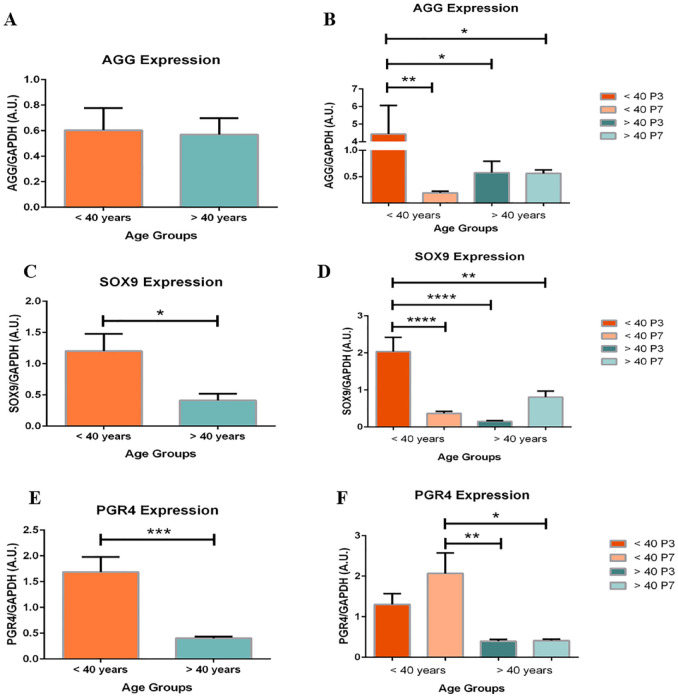
Age-based expression of aggrecan (AGG) expression (**A** and **B**) and SOX9 (**C** and **D**), and PGR4 (**E** and **F**). (**A**) Similar levels of AGG expression between age groups (n=6). (**B**) AGG expression is significantly decreased in P7 compared to P3 chondrocytes within <40 age group (23-fold change; *P* < .01; n = 3). (**C**) Pooled chondrocytes in the >40 age group express significantly less SOX9 than chondrocytes in the <40 age group (2.9-fold change; *P* < .05; n = 6). (**D**) SOX9 expression is significantly decreased in P7 compared to P3 chondrocytes within <40 age group (5.6-fold change; *P* < .001; n = 3). (**E**) Pooled chondrocytes in the >40 age group express significantly less PGR4 than chondrocytes in the <40 age group (*P* < .001; n = 6). (**F**) P7 chondrocytes in the >40 age group express significantly less PGR4 than age contrasted P7 chondrocytes (*P* < .05; n = 3). Data are presented as the mean ± SEM.

No significant difference was found between age groups after analysis of pooled chondrocytes. However, **Figure 4B** shows a significant 23-fold decrease in AGG expression within the <40 group between P3 and P7 (*P* < .01). No significant difference was found within the >40 group.

Chondrocytes in the >40 groups expressed significantly less SOX9 than chondrocytes in the <40 age group (**Fig. 4C**; 2.9-fold change; *P* < .05). Similarly, the expression of SOX9 showed a significant 5.6-fold decrease between P3 and P7 of the <40 group (**Fig. 4D**; *P* < .01).

PGR4 expression also differed between and within age groups. **Figure 4E** and **F** show pooled chondrocytes in the >40 groups express significantly less PGR4 compared to the <40 age group (*P* < .001). In addition, significant differences exist in PGR4 expression between age groups when subdivided into passages (*P* < .05). When comparing PGR4 expression of P7 chondrocytes between the age groups, the P7 chondrocytes in the >40 group expressed significantly less PGR4 than <40 chondrocytes (*P* < .05).

### COL1 and P53 Expression, and Maintenance of BMP4 Expression between Age Groups

**Figure 5.**
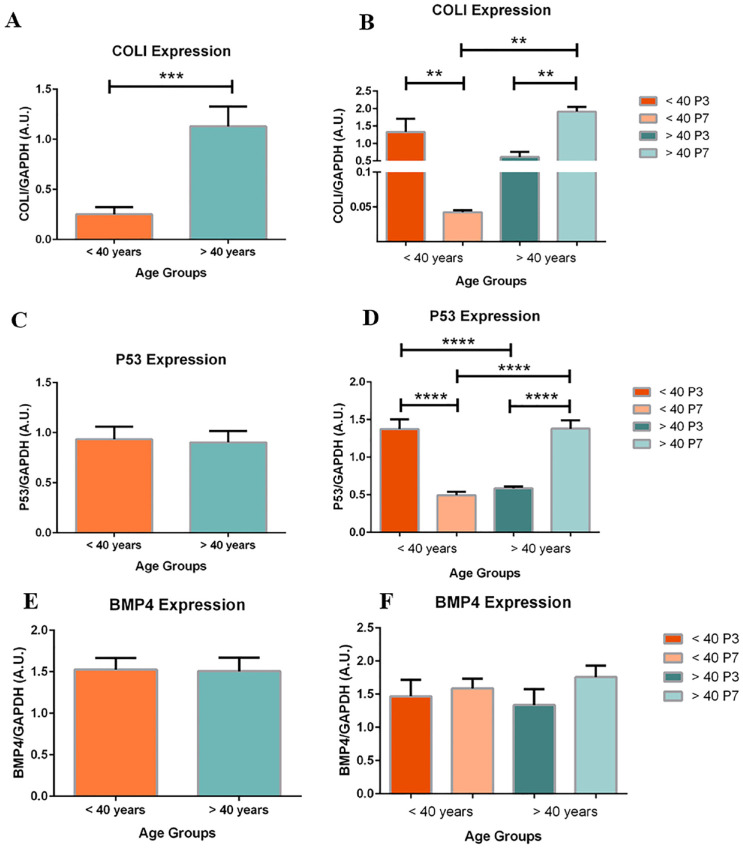
Age-based expression of COLI (**A** and **B**), P53 (**C** and **D**), and BMP4 (**E** and **F**). (**A**) COLI expression of pooled chondrocytes is significantly increased in the >40 age group compared to the <40 age group (*P* < .001; n = 6). (**C**) No significant difference in P53 expression was found between age groups (n = 6). (**B**, **D**) COLI and P53 expression is significantly decreased in P7 chondrocytes compared to P3 chondrocytes in the <40 age group (*P* < .01; n = 3). In the >40 age group, COLI and P53 expression is significantly increased in P7 chondrocytes compared to P3 (*P* < .01; n = 3). BMP4 expression is similar between age groups. (**E**) Pooled chondrocytes samples showed no significant difference in BMP4 expression between age groups (*P* = .9; n = 6). (**F**) Stratified by passage, no significant differences were found within age group or between (n = 3). Data are presented as the mean ± SEM.

COLI and P53 expression demonstrated an inverse relationship between age groups. In the <40 age group, COLI (33-fold change; *P* < .01) and P53 (2.8-fold change; *P* < .001) expression significantly decreased when comparing P7 to P3 chondrocytes (**Fig. 5B** and **D**). Conversely, in the >40 age group, changes in expression for COLI and P53 were inverted. COLI (3.2-fold change; *P* < .01) and P53 (2.4-fold change; *P* < .001) expression significantly increased when comparing P7 to P3 chondrocytes (**Fig. 5B** and **D**).

Subdivided by passage, changes in the expression of COLI and P53 in the <40 group were similar to profiles of dedifferentiating chondrocytes, while changes in expression in the >40 group were resembling to profiles of mature articular chondrocytes.

When stratified by passage number and pooled between groups, no significant differences were found in BMP4 expression (**Fig. 5E** and **F**; *P* = .9).

### Sex Comparison for CPM Gene Expression

When assessed by sex, significant changes in COLI, AGG, and SOX9 expression demonstrate dedifferentiation of chondrocytes from male patients compared to female.

**Figure 6.**
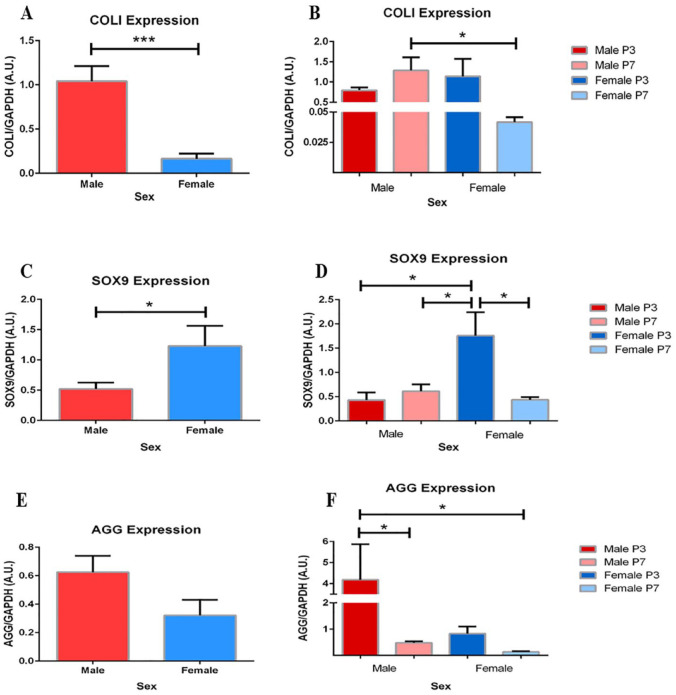
Greater expression of COLI in male chondrocytes. (**A**) Pooled male chondrocytes exhibit a 6.5-fold greater expression of COLI compared to pooled female chondrocytes (*P* < .001; n = 6). (**B**) Subdivided by passage, P7 male chondrocytes exhibit a 32-fold greater expression of COLI compared to P7 female chondrocytes (*P* < .05; n = 3). Male chondrocytes express significantly less SOX9 than female chondrocytes. (**C**) Overall expression of SOX9 is significantly less in male chondrocytes compared to female chondrocytes (*P* < .05; n = 6). (**D**) Male P3 and P7 chondrocytes express significantly less SOX9 than female P3 chondrocytes (*P* < .05; n = 3). SOX9 expression significantly decreases from P3 to P7 chondrocytes within the female group (*P* < .05; n = 3). Sex differences in aggrecan (AGG) expression exist only within group. (**E**) No significant difference in AGG expression exists between sexes (n = 6). (**F**) Male P7 chondrocytes express significantly less AGG compared to male P3 (8.9-fold change; *P* < .05; n = 3). Male P3 chondrocytes also expresses significantly more AGG than female P7 chondrocytes (32-fold change; *P* < .05; n = 3). Data are presented as the mean ± SEM.

COLI expression in pooled male chondrocytes was 1.6-fold greater than pooled female chondrocytes (**Fig. 6A**; *P* < .001). Subdivided by passage, male P7 chondrocytes express significantly more COLI than female P7 chondrocytes (**Fig. 6B**; 32-fold change; *P* < .05); however, no significant difference was found within each sex group.

Similarly, pooled samples of male chondrocytes express significantly less SOX9 than pooled samples of female chondrocytes (**Fig. 6C**; 2.3-fold change; *P* < .05). Both P3 and P7 male chondrocyte also express significantly less SOX9 than female P3 chondrocytes. Specifically, male P3 chondrocytes exhibit a 4.1-fold lesser expression of SOX9 than female P3 chondrocytes (**Fig. 6D**; *P* < .05), and male P7 chondrocytes exhibit a 2.9-fold lesser expression of SOX9 than female P3 chondrocytes (**Fig. 6D**; *P* < .05). Female chondrocytes exhibit a within-group difference, as SOX9 expression decreases 4.1-fold from P3 to P7 (**Fig. 6C** and **D**; *P* < .05).

Finally, observable and significant differences in AGG expression exist between and within sex groups, respectively (**Fig. 6E** and **F**). Although not significant, female chondrocytes express 1.9-fold less AGG than males. This change may be impacted by the significantly less AGG expression in P7 female chondrocytes compared to P3 male chondrocytes (**Fig. 6F**; *P* < .005). Within the male group, P7 chondrocytes expresses 8.9-fold less AGG compared to P3 chondrocytes, highlighting a change in ECM expression (*P* < .05).

### Expression of BMP4, P53, and PGR4 between Sexes

**Figure 7.**
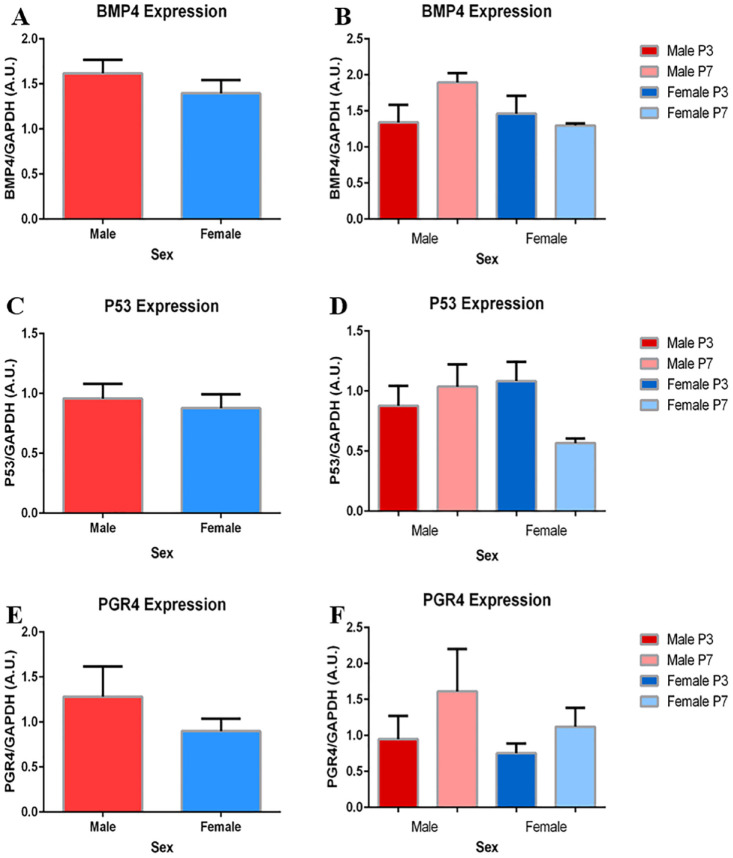
No significant differences in BMP4, P53, or PGR4 expression exist between sexes. (**A**, **B**) Represent the pooled and subdivided sex comparisons for BMP4 (*P* = .3). (**C**, **D**) Represent the pooled and subdivided sex comparisons for P53 (*P* = .6). (**E**, **F**) Represent the pooled and subdivided sex comparisons for PGR4 (*P* = .3). Data are presented as the mean ± SEM. **A**, **C**, **E** represent a sample size of 6. **B**, **D**, **E** represent a sample size of 3.

Comparisons between sexes for pooled and subdivided data did not reach significance in expression of BMP4, P53, or PGR4. From a passage perspective, expression of BMP4, P53, and PGR4 were maintained from P3 to P7 (**Fig. 7A-F**).

## Discussion

### General Comments

Based on guideline of the *Primer Verification* listed in the Materials and Methods section, COLII measurements were excluded from analysis. This is considered to be acceptable for 3 reasons: (1) there are accepted, alternative CPMs for mature chondrocytes including AGG which was studied, (2) all fluorescent expressions were standardized to GAPDH and the resulting limited expression of COLII would not allow for significant analysis, and (3) considering the relationship between SOX9 and COLII, the limited expression of COLII may be a factor of the limited SOX9 expression and therefore, a potential correlation.

### Comparison of CPM Gene Expression between Passages

AGG is one of many proteoglycans critical for proper functioning of articular cartilage. The function of AGG specifically is characterized by its ability to link hyaluronan, forming large proteoglycan aggregates. These aggregates fill the intrafibrillar space, providing mature articular cartilage with the osmotic pressure needed to resist compressive forces.^26^ Of the 6 CPM genes tested, significant differences were found only in expression of AGG between P3 and P7 (**Fig. 6A**).

AGG gene expression decreased 7.4-fold in P7 cells compared to P3, suggesting lesser proteoglycans in P7 cultures and possibly the dedifferentiation of chondrocytes. As chondrocytes grow to confluence and progress through passages, changes in phenotypic gene expression can be accepted mainly as a result in change of the extracellular environment or niche.^18,19^ As a result, proteoglycan gene expression is higher in early chondrocyte passages in efforts to secrete more matrix and establish a suitable environment for growth and function. Thus, the literature has demonstrated the presence of AGG, and measurements of a high ratio of AGG to versican, characteristic of mature articular chondrocytes.^21^

In addition to AGG, non-significant yet observable differences in ECM factors and components may provide support for chondrocyte dedifferentiation, although contradictions exist. Beginning with collagen fibers, the expression of COLII/COLI is an alternative, accepted ratio characterizing mature articular cartilage.^21,27^ In this study, COLII measurements were excluded due to indiscernible fluorescence expression and no significant changes in COLI expression were observed between passages (**Fig. 6B**). However, observable changes in SOX9 expression are worth noting.

As mentioned earlier, SOX9 is a transcription factor required for the expression of the COLII gene, characterizing condensation and maturation of articular cartilage chondrocytes.^28,29^ Although not significant, the 2-fold decrease in SOX9 expression in P7 chondrocytes may suggest a decrease in COLII production and a relatively lower ratio of COLII/COLI (**Fig. 6E**).

Conversely, the maintenance of P53 and BMP4 expression suggests that dedifferentiation of chondrocytes throughout passaging is much less likely. P53 is a tumor suppressor gene functioning to halt the induction of pluripotent cells.^29^ Without significant change in P53 gene expression, relative suppression levels of stem-like cell potential should remain constant, limiting the reversion of chondrocytes. Contrary to the interpretation of P53, BMP4 expression marks the acceleration of chondrocyte differentiation and maturation.

In addition, observable increases in PGR4 gene expression contradict the interpretation of AGG expression. Referring to a group of proteoglycans, the SZP is a major component of the PGR4 group. Produced exclusively by specialized chondrocytes in the superficial zone of articular cartilage, measurements of SZP/PGR4 expression suggest the presence of mature, superficial articular chondrocytes.^30^ A 1.6-fold increase in PGR4 expression may suggest the increase in SZP protein content and the maturation of P7 chondrocytes instead of their dedifferentiation. Overall, the results suggest that cultured articular chondrocytes maintain CPM gene expression and reserve their phenotype up to P7.

### Comparison of CPM Gene Expression between Age Groups

Each gene expression profile was compared strictly between age groups, then subdivided by passage to account for significant or influential differences such as AGG gene expression. Similar to passage comparisons, interpretation of CPM genes alluded to similar signs of dedifferentiation, contradicting expression, or relative levels of expression between age groups.

Although not significant in pooled data, in the <40 group, P7 chondrocytes expressed 23-fold less AGG than P3 chondrocytes (**Fig. 4B**). In addition, SOX9 and PGR4 expression was significantly lower in the >40 group than the <40 group (**Fig. 4D** and **F**). Interpreting first the relationship between SOX9 and PGR4, a decreased expression in both is indicative of dedifferentiated chondrocytes. The lack of transcription factor SOX9 will limit the production of essential COLII, and the absence of PGR4 may correlate to the absence of SZP; a maturely expressed protein. This loss of proteoglycan content, possibly chondrogenic potential from early effects of passaging, mimics the effects of aging by inhibiting proper aggregation and disrupting the collagen network.^31^ Decreases in hydraulic and osmotic pressure experienced by the tissue *in vivo* may than result subsequently increasing the permeability of the cartilage and hindering its resistance to compressive forces in a fashion similar to OA.^32,33^

However, age-based expression of COLI and P53 present contradicting results, limiting the interpretation of individual gene expression as markers of differentiation. In the <40 group, COLI expression decreased 33-fold, and P53 decreased 2.8-fold from P3 to P7 (**Fig. 5B** and **D**). In this case, a decrease in COLI expression would suggest maturation of chondrocytes, whereas a decrease in P53 would also suggest dedifferentiation of chondrocyte through suppression release. Conversely, in the >40 group, COLI expression increased 3.2-fold and P53 expression increased 2.4-fold from P3 to P7 (**Fig. 5A**). Together, these results indicate that passaging chondrocytes from younger and older donors differ in the direction of their phenotypic change. The early removal of suppression seen in P53 may suggest a compensatory mechanism in the younger population, allowing chondrocyte and ECM turnover.^33^

Finally, BMP4 gene expression did not change within, nor was there a significant difference between age groups (**Fig. 5E** and **F**). Thus, chondrocytes isolated from younger and older participants express similar levels of BMP4, suggesting similar levels of condensation and maturation with aging. Overall, the results indicate that younger chondrocytes may be more likely to dedifferentiate, offering greater conservation of CPM expression.

### Comparison of CPM Gene Expression between Sexes

Stemming from the prior discussion on cartilage disease and injury repair, OA is known to affect significantly more females than males over the age of 60.^34,35^ Considering age as the primary risk factor for OA, and sex as secondary, there are 2 important factors to acknowledge before interpreting sex results. First, it is important to note and integrate the prior relationships discovered in age-based analyses in disease interpretation of sex results. Second, sex differences should also be viewed as an independent variable, with summed results minimizing the effect of both passage and age.

The established ratios expressing mature chondrocytes such as COLII/COLI, and the relationship between SOX9 and COLII expression are highlighted in sex-based comparisons of chondrocytes. Male chondrocytes express significantly more COLI (**Fig. 6A**) and less SOX9 (**Fig. 6C**) than female chondrocytes, complementing dedifferentiation of male chondrocytes. Recalling that a high COLII/COLI ratio is characteristic of mature, differentiation chondrocytes, an increased COLI expression would directly lower the ratio and mark dedifferentiation [18, 24]. Although COLII was not directly measured, increased expression or activation of the SOX9 gene is an early, imperative step in the expression of COLII.^28^

On the contrary, significant changes in AGG and lack of change in remaining genes further limit the evaluation of chondrogenic potential between sexes. Building from the expression of COLI and SOX9, male chondrocytes would be expected to have less AGG than female chondrocytes, further supporting dedifferentiation. In **Figure 6F**, male chondrocyte expression of AGG was shown to significantly decrease from P3 to P7 (8.9-fold change). In addition, summed data show a noticeably greater, though not significant, expression of AGG in males than females. Decreased expression of AGG in higher passaged, older chondrocytes may help to explain the initial drop off in AGG expression seen in P7, though the overall higher mean remains contradictory.

Finally, the lack of significant difference between sexes in BMP4, P53, and PGR4 expression can be interpreted as chondrogenic potential similarities between sexes. Taken together, the differences presented in COLI and SOX9 between sexes may suggest the retention of chondrogenic potential in male chondrocytes. In relation to function and disease, the ability of male chondrocytes to dedifferentiate and alter phenotype may present a compensatory mechanism. Having such a mechanism in place may aid in the injury repair cycle, the restoration of lost proteoglycans, and the production of COLII and SZP, preserving cartilage function. Thus, in association with age results, older female chondrocytes may be more prone to dysfunction and disease due to their loss of CPMs.

### Limitations and Future Directions

Small sample size is one factor that may pose limitation to these study results. In addition to being subdivided into small group comparisons (n = 3) based on age and sex, the limited number of samples overall may skew the finding of significance. Although a total of 6 cell cultures is a suitable number for analysis, an increase in overall sample number or employment of alternative techniques may help to confirm the findings.

The overall extent of the analysis performed should be extended to confirm cell type and composition, as well as specific protein levels. Expression of selected CPMs present only in chondrocyte populations is a crucial aspect of this study, suggesting the isolation of chondrocytes in culture. However, a histological examination of chondrocytes paired with immunohisto-chemically staining techniques would provide foundational support in addition to the collagenase isolation for the purity of the chondrocyte cultures.

Despite these limitations, there are some potential future applications for these results. Focused primarily on cartilage tissue engineering, the characterization of passage, age, and sex differences may provide an aid for optimizing chondrocyte isolation, freezing, and culturing techniques. Prior studies have attempted to optimize cartilage tissue engineering, culturing autologous chondrocytes up to 4 weeks before successful implementation.^5^ In addition, the same research group identified the approximate number of cells needed to engineer specifically sized tissue.^4^ Together, the culturing of only 20,000 cells was efficient in producing a 3 cm^2^ cartilage construct, expressed mature cartilage genes, and became biomechanically sound.

Integrating results of this study, passage-based analysis would suggest that isolated chondrocytes could grow and passage up to 7 times without expressing signs of dedifferentiation or loss of crucial cartilage phenotype. The maintenance of chondrogenic potential may allow researchers and clinicians more variability and growth time, optimizing the type of tissues for implantation regardless of cell count or final size. However, younger, male chondrocytes are more likely to dedifferentiate compared to older, female chondrocytes, favoring their use in the culturing, engineering, and implantation process. Taken together, researchers and clinicians can make more sound judgments in isolating chondrocytes, opting to freeze younger chondrocytes for future implantation.

## Conclusion

The results and subsequent discussion suggest there is maintenance of CPMs throughout passages, and loss of chondrogenic potential in female and older chondrocytes. After analysis of 6 CPM genes, only AGG gene expression decreased from P3 to P7, suggesting the maintenance of chondrogenic potential up to P7. In addition, changes in AGG, SOX9, and PGR4 expression between age groups suggest that chondrocytes will lose their chondrogenic potential with age. Although significant differences in remaining genes were not found, the interplay between AGG, SOX9, and PGR4 align with known changes in aging and osteoarthritic cartilage. Finally, sex-based analysis suggests that chondrogenic potential is not conserved between sexes. Specifically, significant changes in COLI and SOX9 expression present a profile of dedifferentiation in cultured male chondrocytes.

Despite the limitations of the study, interpretations of the results reflect careful consideration for confounding factors and provide a step forward in the characterization of chondrogenic potential in culture. Researchers and clinicians could use resulting considerations of passage, age, and sex differences to better inform and optimize methods for isolation, culturing, and engineering of autologous cartilage structures.

## Supplemental Material

sj-pptx-1-car-10.1177_19476035241241930 – Supplemental material for Expression of Chondrogenic Potential Markers in Cultured Chondrocytes from the Human Knee Joint Supplemental material, sj-pptx-1-car-10.1177_19476035241241930 for Expression of Chondrogenic Potential Markers in Cultured Chondrocytes from the Human Knee Joint by John-Peter Bonello, M. Yat Tse, Trevor J. G. Robinson, Davide D. Bardana, Stephen D. Waldman and Stephen C. Pang in CARTILAGE

## References

[1] Hunziker EB . Articular cartilage repair: are the intrinsic biological constraints undermining this process insuperable? Osteoarthritis Cartilage. 1999 Jan;7(1):15-28. doi:10.1053/joca.1998.0159 10367012

[2] Brittberg M Lindahl A Nilsson A Ohlsson C Isaksson O Peterson L . Treatment of deep cartilage defects in the knee with autologous chondrocyte transplantation. N Engl J Med. 1994 Oct 6;331(14):889-95. doi:10.1056/NEJM199410063311401 8078550

[3] De Bari C Dell’Accio F Tylzanowski P Luyten FP . Multipotent mesenchymal stem cells from adult human synovial membrane. Arthritis Rheum. 2001 Aug;44(8):1928-42. doi:10.1002/1529-0131(200108)44:8<1928::AID-ART331>3.0.CO;2-P 11508446

[4] Brenner JM Kunz M Tse MY Winterborn A Bardana DD Pang SC , et al Development of large engineered cartilage constructs from a small population of cells. Biotechnol Prog. 2013 Jan-Feb;29(1):213-21. doi:10.1002/btpr.1670 23197468

[5] Brenner JM Ventura NM Tse MY Winterborn A Bardana DD Pang SC , et al Implantation of scaffold-free engineered cartilage constructs in a rabbit model for chondral resurfacing. Artif Organs. 2014 Feb;38(2):E21-32. doi:10.1111/aor.12199 24571514

[6] Al Faqeh H Nor Hamdan BM Chen HC Aminuddin BS Ruszymah BH . The potential of intra-articular injection of chondrogenic-induced bone marrow stem cells to retard the progression of osteoarthritis in a sheep model. Exp Gerontol. 2012 Jun;47(6):458-64. doi:10.1016/j.exger.2012.03.018 22759409

[7] Chiari C Koller U Dorotka R Eder C Plasenzotti R Lang S , et al A tissue engineering approach to meniscus regeneration in a sheep model. Osteoarthritis Cartilage. 2006 Oct;14(10):1056-65. doi:10.1016/j.joca.2006.04.007 16731009

[8] Jiang T Xu G Wang Q Yang L Zheng L Zhao J , et al In vitro expansion impaired the stemness of early passage mesenchymal stem cells for treatment of cartilage defects. Cell Death Dis. 2017 Jun 1;8(6):e2851. doi:10.1038/cddis.2017.215 PMC5520885 28569773

[9] Murtaugh LC Chyung JH Lassar AB . Sonic hedgehog promotes somitic chondrogenesis by altering the cellular response to BMP signaling. Genes Dev. 1999 Jan 15;13(2):225-37. doi:10.1101/gad.13.2.225 PMC316396 9925646

[10] Lefebvre V Smits P . Transcriptional control of chondrocyte fate and differentiation. Birth Defects Res C Embryo Today. 2005 Sep;75(3):200-12. doi:10.1002/bdrc.20048 16187326

[11] Cancedda R Descalzi Cancedda F Castagnola P . Chondrocyte differentiation. Int Rev Cytol. 1995;159:265-358. doi:10.1016/s0074-7696(08)62109-9 7737795

[12] Valcourt U Gouttenoire J Aubert-Foucher E Herbage D Mallein-Gerin F . Alternative splicing of type II procollagen pre-mRNA in chondrocytes is oppositely regulated by BMP-2 and TGF-beta1. FEBS Lett. 2003 Jun 19;545(2-3):115-9. doi:10.1016/s0014-5793(03)00510-6 12804760

[13] Goldring MB Tsuchimochi K Ijiri K . The control of chondrogenesis. J Cell Biochem. 2006 Jan 1;97(1):33-44. doi:10.1002/jcb.20652 16215986

[14] Lefebvre V Behringer RR de Crombrugghe B . L-Sox5, Sox6 and Sox9 control essential steps of the chondrocyte differentiation pathway. Osteoarthritis Cartilage. 2001;9(Suppl A):S69-75. doi:10.1053/joca.2001.0447 11680692

[15] Huang W Chung UI Kronenberg HM de Crombrugghe B . The chondrogenic transcription factor Sox9 is a target of signaling by the parathyroid hormone-related peptide in the growth plate of endochondral bones. Proc Natl Acad Sci USA. 2001 Jan 2;98(1):160-5. doi:10.1073/pnas.98.1.160 PMC14561 11120880

[16] MacLean HE Kim JI Glimcher MJ Wang J Kronenberg HM Glimcher LH . Absence of transcription factor c-Maf causes abnormal terminal differentiation of hypertrophic chondrocytes during endochondral bone development. Dev Biol. 2003 Oct 1;262(1):51-63. doi:10.1016/s0012-1606(03)00324-5 14512017

[17] Gerber HP Vu TH Ryan AM Kowalski J Werb Z Ferrara N . VEGF couples hypertrophic cartilage remodeling, ossification and angiogenesis during endochondral bone formation. Nat Med. 1999 Jun;5(6):623-8. doi:10.1038/9467 10371499

[18] Kuhn NZ Tuan RS . Regulation of stemness and stem cell niche of mesenchymal stem cells: implications in tumorigenesis and metastasis. J Cell Physiol. 2010 Feb;222(2):268-77. doi:10.1002/jcp.21940 19847802

[19] Tintut Y Alfonso Z Saini T Radcliff K Watson K Boström K , et al Multilineage potential of cells from the artery wall. Circulation. 2003 Nov 18;108(20):2505-10. doi:10.1161/01.CIR.0000096485.64373.C5 14581408

[20] Gleizes PE Munger JS Nunes I Harpel JG Mazzieri R Noguera I , et al TGF-beta latency: biological significance and mechanisms of activation. Stem Cells. 1997;15(3):190-7. doi:10.1002/stem.150190 9170210

[21] Jakob M Demarteau O Schafer D Hintermann B Dick W Heberer M , et al Specific growth factors during the expansion and redifferentiation of adult human articular chondrocytes enhance chondrogenesis and cartilaginous tissue formation in vitro. J Cell Biochem. 2001 Mar 26;81(2):368-77. doi:10.1002/1097-4644(20010501)81:2<368::aid-jcb1051>3.0.co;2-j 11241676

[22] Waldman SD Usmani Y Tse MY Pang SC . Differential effects of natriuretic peptide stimulation on tissue-engineered cartilage. Tissue Eng Part A. 2008 Mar;14(3):441-8. doi:10.1089/tea.2007.0035 18333796

[23] Robinson TJ . Developing protocols for the molecular and morphological characterization of human articular cartilage and tissue engineered cartilage. Queen’s University, Kingston, Ontario, Canada; 2012.

[24] Pang SC . In vitro proliferation of aortic smooth muscle cells from spontaneously hypertensive and normotensive rats. J Pathol. 1989 Jun;158(2):167-73. doi:10.1002/path.1711580212 2754547

[25] Waldbilling DK Pang SC . Differential proliferation of rat aortic and mesenteric smooth muscle cells in culture. Histol Histopathol. 1992 Apr;7(2):199-207. 1515702

[26] Sophia Fox AJ Bedi A Rodeo SA . The basic science of articular cartilage: structure, composition, and function. Sports Health. 2009 Nov;1(6):461-8. doi:10.1177/1941738109350438 PMC3445147 23015907

[27] Diaz-Romero J Nesic D Grogan SP Heini P Mainil-Varlet P . Immunophenotypic changes of human articular chondrocytes during monolayer culture reflect bona fide dedifferentiation rather than amplification of progenitor cells. J Cell Physiol. 2008 Jan;214(1):75-83. doi:10.1002/jcp.21161 17559082

[28] Akiyama H Chaboissier MC Martin JF Schedl A de Crombrugghe B . The transcription factor Sox9 has essential roles in successive steps of the chondrocyte differentiation pathway and is required for expression of Sox5 and Sox6. Genes Dev. 2002 Nov 1;16(21):2813-28. doi:10.1101/gad.1017802 PMC187468 12414734

[29] Olivos DJ Mayo LD . Emerging non-canonical functions and regulation by p53: p53 and stemness. Int J Mol Sci. 2016 Nov 26;17(12):1982. doi:10.3390/ijms17121982 27898034 PMC5187782

[30] Schumacher BL Hughes CE Kuettner KE Caterson B Aydelotte MB . Immunodetection and partial cDNA sequence of the proteoglycan, superficial zone protein, synthesized by cells lining synovial joints. J Orthop Res. 1999 Jan;17(1):110-20. doi:10.1002/jor.1100170117 10073655

[31] Pearle AD Warren RF Rodeo SA . Basic science of articular cartilage and osteoarthritis. Clin Sports Med. 2005 Jan;24(1):1-12. doi:10.1016/j.csm.2004.08.007 15636773

[32] Akizuki S Mow VC Muller F Pita JC Howell DS . Tensile properties of human knee joint cartilage. II. Correlations between weight bearing and tissue pathology and the kinetics of swelling. J Orthop Res. 1987;5(2):173-86. doi:10.1002/jor.1100050204 3572588

[33] Akkiraju H Nohe A . Role of chondrocytes in cartilage formation, progression of osteoarthritis and cartilage regeneration. J Dev Biol. 2015 Dec;3(4):177-92. doi:10.3390/jdb3040177 PMC4916494 27347486

[34] Felson DT Naimark A Anderson J Kazis L Castelli W Meenan RF . The prevalence of knee osteoarthritis in the elderly. The Framingham osteoarthritis study. Arthritis Rheum. 1987 Aug;30(8):914-8. doi:10.1002/art.1780300811 3632732

[35] Zhang Y Jordan JM . Epidemiology of osteoarthritis. Clin Geriatr Med. 2010 Aug;26(3):355-69. doi:10.1016/j.cger.2010.03.001 PMC2920533 20699159

