# Supplementary material for: Expression of Chondrogenic Potential Markers in Cultured Chondrocytes from the Human Knee Joint
Source: Cartilage. 2024 Apr 14;16(4):518–30. doi: 10.1177/19476035241241930 (PMC11569588; doi:10.1177/19476035241241930)
Supplement: sj-pptx-1-car-10.1177_19476035241241930 – Supplemental material for Expression of Chondrogenic Potential Markers in Cultured Chondrocytes from the Human Knee Joint [file sj-pptx-1-car-10.1177_19476035241241930.pptx]

## Slide 1
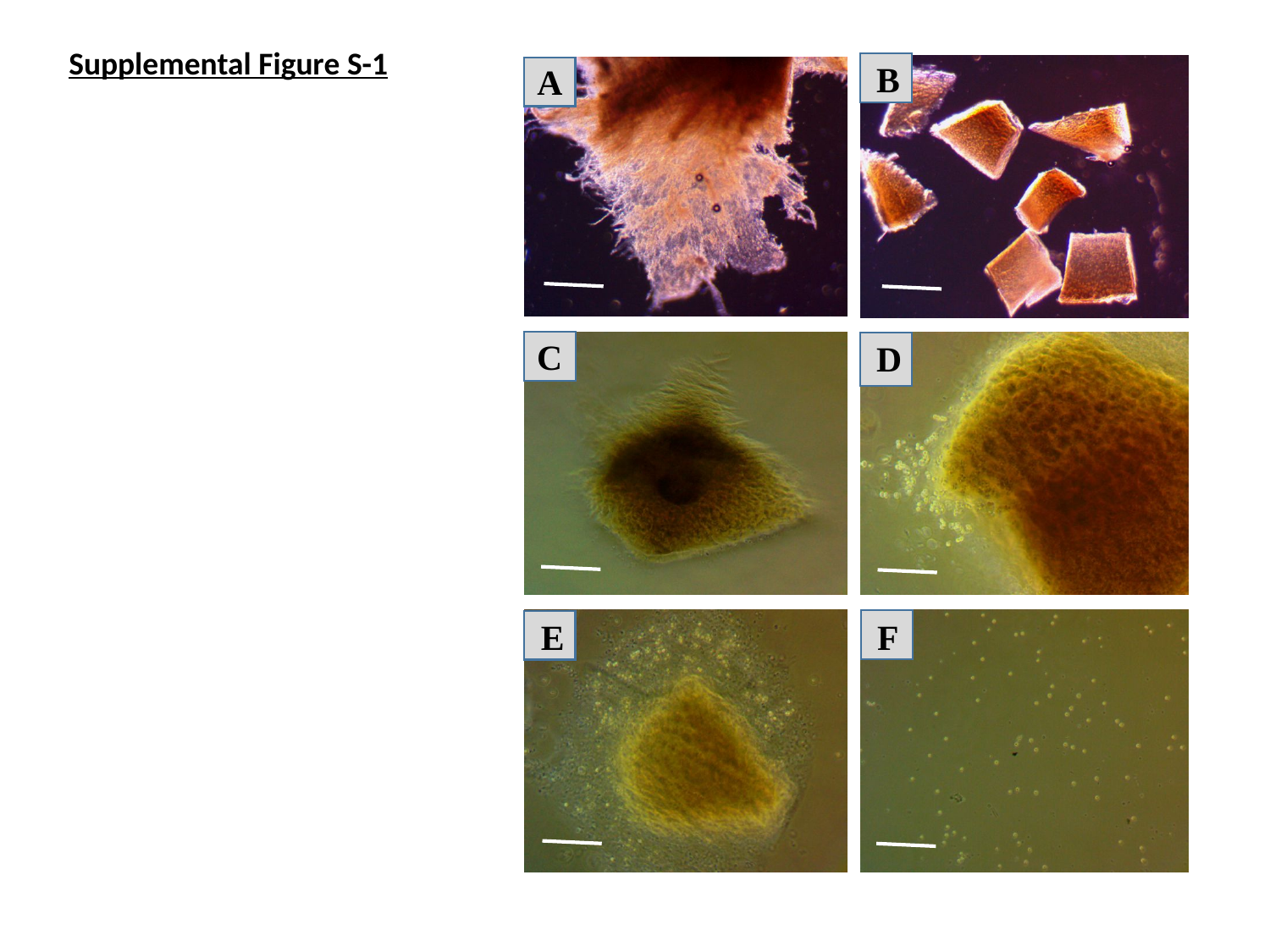

Supplemental Figure S-1
B
A
C
D
F
E

## Slide 2
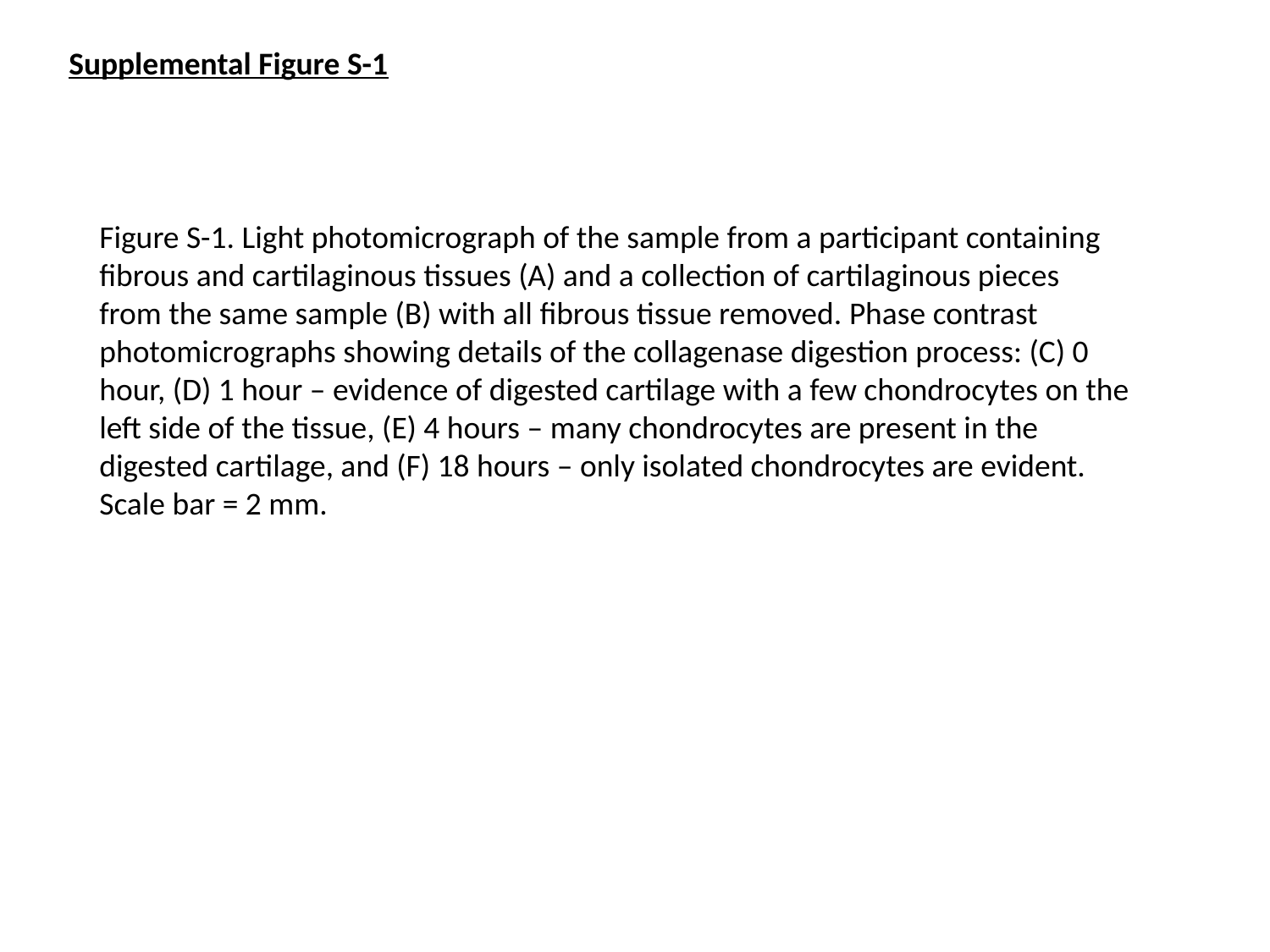

Supplemental Figure S-1
Figure S-1. Light photomicrograph of the sample from a participant containing fibrous and cartilaginous tissues (A) and a collection of cartilaginous pieces from the same sample (B) with all fibrous tissue removed. Phase contrast photomicrographs showing details of the collagenase digestion process: (C) 0 hour, (D) 1 hour – evidence of digested cartilage with a few chondrocytes on the left side of the tissue, (E) 4 hours – many chondrocytes are present in the digested cartilage, and (F) 18 hours – only isolated chondrocytes are evident. Scale bar = 2 mm.
